# Global and Gender Equity in Oligodendroglioma Research: A Comprehensive Bibliometric Analysis Following the COVID-19 Pandemic

**DOI:** 10.7759/cureus.51161

**Published:** 2023-12-27

**Authors:** Kashish Malhotra, Mert Marcel Dagli, Jaskeerat Gujral, Gabrielle Santangelo, Kashish Goyal, Connor Wathen, Ali K Ozturk, William C Welch

**Affiliations:** 1 Department of Surgery, Dayanand Medical College and Hospital, Ludhiana, IND; 2 Institute of Applied Health Research, University of Birmingham, Birmingham, GBR; 3 Department of Neurosurgery, Perelman School of Medicine, University of Pennsylvania, Philadelphia, USA; 4 Department of Internal Medicine, Dayanand Medical College and Hospital, Ludhiana, IND

**Keywords:** oligodendroglioma, gender equity, global equity, covid-19, bibliometric analysis

## Abstract

Oligodendrogliomas are rare brain tumors arising from oligodendrocytes; there is a limited understanding of their pathogenesis, which leads to challenges in diagnosis, prognosis, and treatment. This study aimed to conduct a comprehensive bibliometric analysis of the oligodendroglioma literature to assess the current state of research, identify research trends, and elucidate implications for future research.

The Lens^®^ database was used to retrieve journal articles related to "oligodendroglioma" without geographic or temporal restrictions. Year-on-year trends in publication and funding were analyzed. Global and gender equity were assessed using the Namsor^® ^Application programming interface. Collaboration patterns were explored using network visualizations. Keyword analysis revealed the most prominent themes in oligodendroglioma research.

Out of 9701 articles initially retrieved, 8381 scholarly journal articles were included in the final analysis. Publication trends showed a consistent increase until 2020, followed by a sharp decline likely due to the COVID-19 pandemic. Global representation revealed researchers from 86 countries, with limited participation from low and middle-income countries (LMICs). Gender inequity was evident, with 78.7% of researchers being male. Collaboration analysis revealed a highly interconnected research community. Prognosis, genetic aberrations (particularly "IDH" mutations), and therapeutic options (including chemotherapy and radiotherapy) emerged as dominant research themes.

The COVID-19 pandemic impacted oligodendroglioma research funding and publication trends, highlighting the importance of robust funding mechanisms. Global and gender inequities in research participation underscore the need for fostering inclusive collaboration, especially in LMICs. The interconnected research community presents opportunities for knowledge exchange and innovation. Keyword analysis highlights current research trends and a shift to genetic and molecular understanding.

## Introduction and background

Oligodendrogliomas, a relatively rare type of brain tumor, account for approximately 9.4% of all primary brain and central nervous system (CNS) tumors [1]. Oligodendrogliomas arise from oligodendrocytes, which are cells that produce the myelin sheath that surrounds and insulates nerve fibers in the CNS [2]. Despite advances in treatment options such as surgery, radiation therapy, and chemotherapy, the overall survival rate for oligodendrogliomas remains relatively low. The median survival time (MST) for patients with grade II oligodendrogliomas is around 11.6 years, while grade III oligodendrogliomas have an MST of 3.5 years, and the five-year overall survival rate is around 74.9% and 51.1% for grade II and grade III oligodendrogliomas, respectively, with a majority of patients experiencing recurrent tumors [1,3,4].

The pathogenesis of oligodendrogliomas is not completely understood, which contributes to a lack of understanding of pathogenic pathways for these tumors, as well as the potential for misdiagnosis with other types of brain tumors. The identification of specific genetic aberrations and molecular markers in oligodendrogliomas will aid in the classification, prognosis, and treatment of these tumors. Thus, it is pertinent to critically review the existing literature on the topic [5,6].

Current oligodendroglioma management involves a multimodal approach, including personalized surgery, radiotherapy, and chemotherapy. Treatments, tailored to tumor specifics and patient factors, focus on improving survival and symptom relief, with chemotherapy regimens selected based on genetic markers, continually evolving through research for optimal outcomes.

A bibliometric analysis, in contrast to systematic reviews that delve into specific research questions, provides a comprehensive overview of a field by mapping out prevailing trends, collaborative networks, and thematic evolutions [7]. This approach offers valuable insights into the productivity, impact, and visibility of research in a particular area. This type of analysis can be useful in identifying key players and research trends in a particular field, help with identifying gaps in the literature, and assess funding and collaboration.

The objective of this study is to conduct a comprehensive bibliometric analysis of the literature on oligodendrogliomas to critically evaluate the current state of research on this topic.

## Review

Material and methods

Publication and Funding Trends

We used the Lens® database (www.lens.org) to extract all the journal articles pertaining to "scholarly work" related to the search query “oligodendroglioma” without any time period or geographic restrictions [8]. Lens is a comprehensive search database of over 250 million scholarly records that lists articles and metadata from various databases including Microsoft Academic, PubMed, and Crossref. We then identified the funding bodies that are funding oligodendroglioma research and studied year-on-year trends of the published funded literature.

Global and Gender Equity

To study global equity in oligodendroglioma research, we identified the countries of residence of researchers and classified the countries into high-, upper-middle, lower-middle, and low-income countries as per the 2022 World Bank report [9]. Furthermore, we also identified the leading universities and scientific journals contributing most to the oligodendroglioma literature by publishing articles. To study gender equity, we used the Namsor® application programming interface to identify the genders of the top 1000 researchers by total publications (primary criteria) and citations (secondary criteria) with 95% probability [10]. Namsor has been previously used in the scientific literature to study the genders of researchers [11].

Collaboration and Keywords

To study bibliometrics and connections between various categories, analysis was done using Vos Viewer [12]. Network visualization was done to study underlying links between various entities. Density visualization was done to study the impact of an entity within the whole system. Overlay visualization was done to study trends over time. Co-authorship analysis was done using the "full counting method" with "association strength" as a method of normalization to collaboration among the top 1000 authors who have published the most articles. Similarly, to identify the top 1000 keywords, a co-occurrence analysis was done. To study trends over time in the usage of keywords, overlay visualization was done for keywords.

Results

Publication and Funding Trends

**Figure 1:**
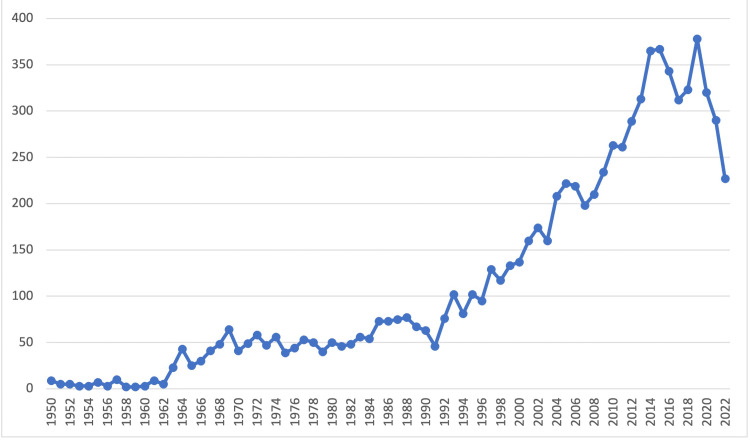
Time Series Line Graph of Annual Oligodendrolioma Publication Volume

A total of 9701 articles were initially extracted in November 2022, but after limiting the search to include only scholarly journal articles, 8381 articles were included in the final analysis. We fetched articles going back to the 1950s and the year-on-year trends in publication showed an overall increasing trend until 2020, which coincided with the onset of the COVID-19 pandemic, as shown in Figure 1.

**Figure 2:**
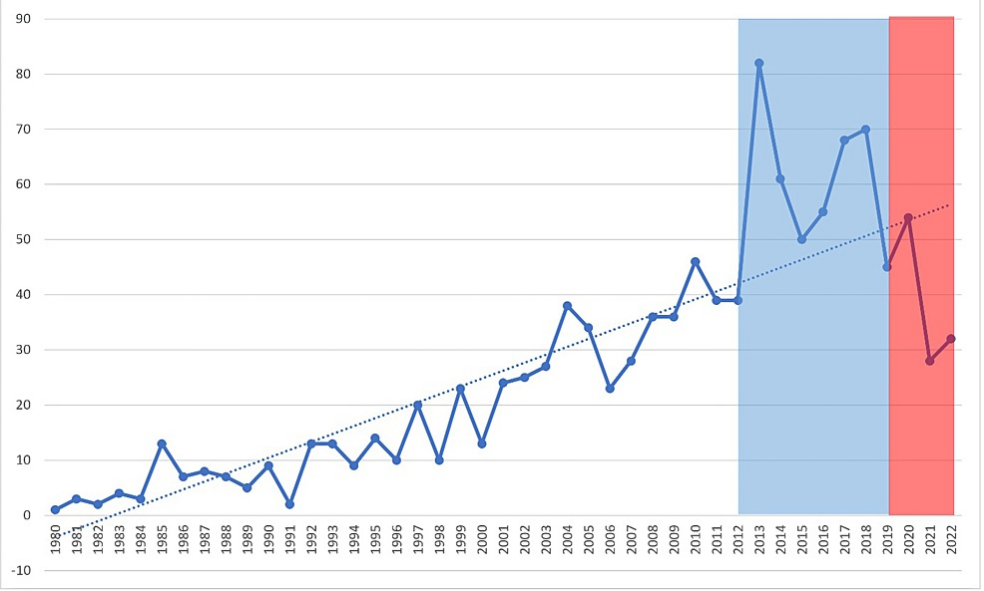
Time Series Line Graph of Funded Annual Oligodendrolioma Publication Volume

Of the 8381 total articles, 2591 (30.9%) were supported by various funding bodies. The top 10 funding bodies by total documents published related to oligodendroglioma research were from high-income countries (HICs) (USA and UK) and one from an upper-middle income country (China), with National Cancer Institute (NCI) NIH HHS leading the list with 571 published documents, followed by National Institute of Neurological Disorders and Stroke (NINDS) NIH HHS with 238 documents, as shown in Table 1. The year-on-year trends of funded literature by these top 10 funding bodies displayed a broad increasing trend with a peak in 2014, followed by a sharp downturn from 2020 to 2022 (Figure 2). This trend in funding closely mirrored the overall publication trends during the same period (Figure 1).

**Table 1:** Top 10 Funding Bodies by Oligodendroglioma Research Publication Volume NCI, National Cancer Institute; NINDS, National Institute of Neurological Disorders and Stroke; NIGMS, National Institute of General Medical Sciences; NCRR, National Center for Research Resources; NICHD, National Institute of Child Health and Human Development; NCATS, National Center for Advancing Translational Sciences

Funding Body	Document Count
NCI NIH HHS	571
NINDS NIH HHS	238
NIGMS NIH HHS	62
Medical Research Council	54
NCRR NIH HHS	48
Cancer Research UK	35
NICHD NIH HHS	34
NCATS NIH HHS	31
National Natural Science Foundation of China	28
Wellcome Trust	27

Global and Gender Inequity

**Figure 3:**
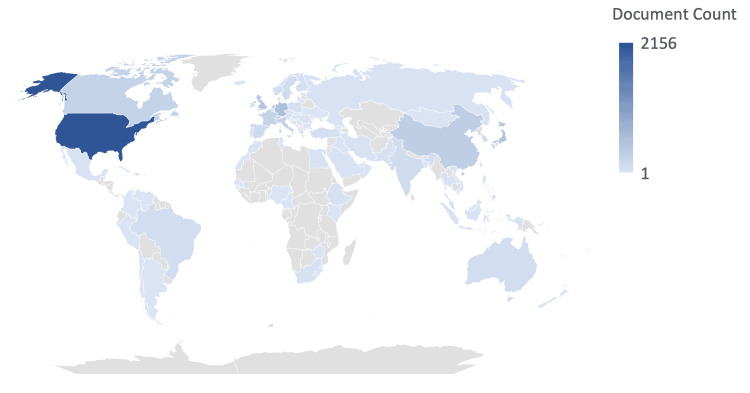
Geographic Publication Volume Distribution Heatmap of Oligodendroglioma Research Authors

Oligodendroglioma researchers resided in 86 countries with extensive involvement from researchers in HICs (USA, Germany, Japan, UK) and some middle-income economies (China, India, Brazil, Turkey) and one low-income economy (Ethiopia) as shown in Figure 3. The institutions and authors with the most published documents on oligodendroglioma are listed in Table 2 and Table 3, respectively. The top 10 authors and institutions were from high-income economies. The journals with the most published articles about oligodendroglioma were Neuro-Oncology, Journal of Neuro-Oncology, and Acta Neuropathologica as shown in Table 4. Out of the 731 scholars whose gender could be determined with 95% probability, a significant majority (78.7%) were male, while only 21.3% were female.

**Table 2:** Top 10 Institutions by Oligodendroglioma Research Publication Volume

Institution Name	Document Count
Harvard University	216
University of California, San Francisco	202
Mayo Clinic	163
University of Texas MD Anderson Cancer Center	153
University of Zurich	137
Memorial Sloan Kettering Cancer Center	131
Erasmus University Rotterdam	125
French Institute of Health and Medical Research	114
Johns Hopkins University	99
Heidelberg University	96

**Table 3:** Top 10 Oligodendroglioma Researchers by Publication Volume

Author Name	Document Count
Michael Weller	85
Martin J van den Bent	84
Andreas von Deimling	81
David N Louis	72
Robert B Jenkins	71
Johan M Kros	70
Dominique Figarella-Branger	61
Marc Sanson	61
Guido Reifenberger	59
Ahmed Idbaih	58

**Table 4:** Top 10 Journals by Publication Volume on Oligodendroglioma

Journal Title	Document Count
Neuro-Oncology	491
Journal of Neuro-Oncology	333
Acta Neuropathologica	193
Journal of Neurosurgery	168
Cancer Research	164
Journal of Neuropathology and Experimental Neurology	146
Neurosurgery	134
Plos One	94
Acta Neurochirurgica	91
Cancer	85

Collaboration Trends

**Figure 4:**
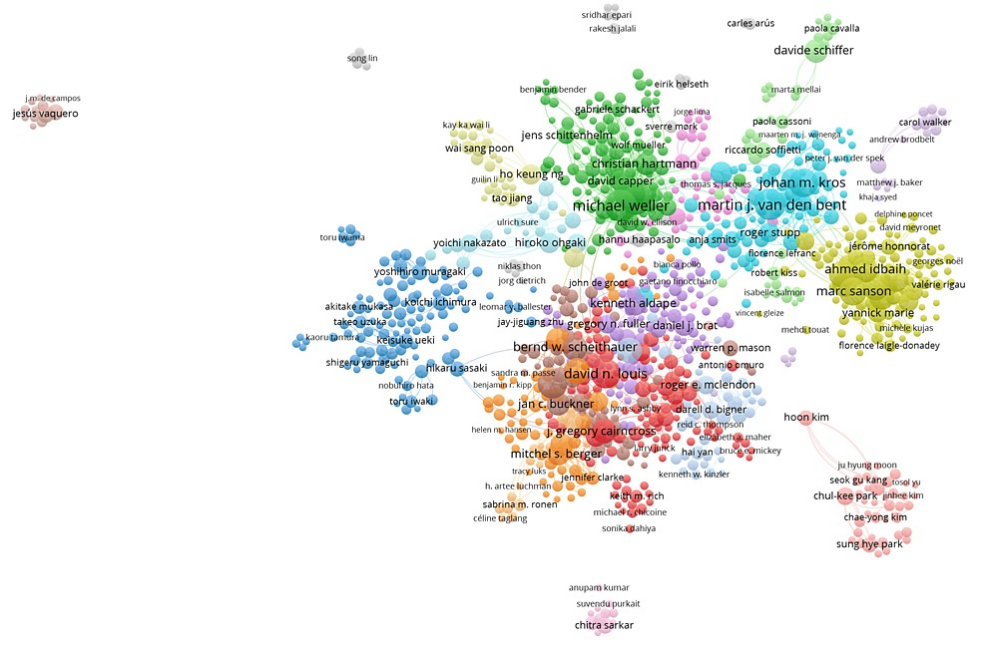
Network Analysis of Oligodendroglioma Researchers

The analysis of collaboration patterns among authors in the field of oligodendroglioma research revealed a highly interconnected community. In the network and density analysis of the top 1000 authors were able to identify 23 clusters with 15099 links (Figure 4).

Keyword Trends

**Figure 5:**
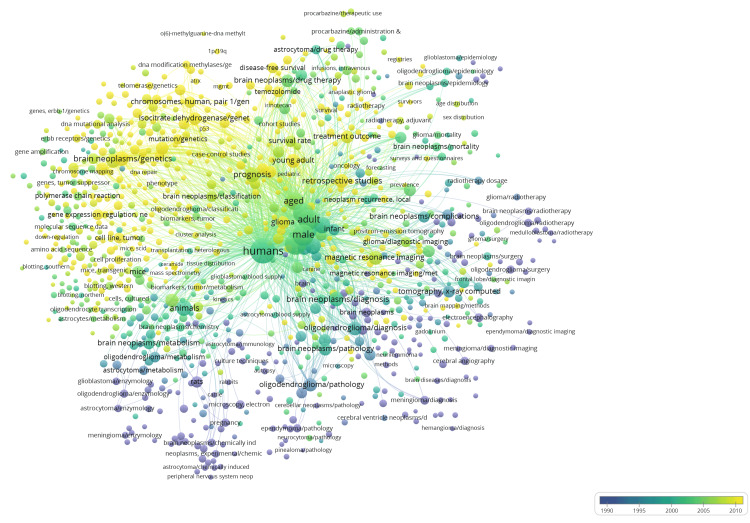
Overlay Keyword Analysis of Oligodendroglioma Research Showing Trends Over Time

Initially we extracted 14561 total keywords of the articles and after excluding general keywords about brain neoplasms, the top 10 keywords related to oligodendroglioma research are shown in Table 5. Of the 4667 author keywords identified, overlay analysis of the top 1000 keywords showed an increased number of articles pertaining to genetic expressions and mutations (Figure 5).

**Table 5:** Top Keywords Pertaining to Oligodendroglioma Research

Keyword	Document Count
Prognosis	67
Idh	49
Chemotherapy	47
Temozolomide	46
Survival	44
1p/19q Codeletion	43
Immunohistochemistry	42
Radiotherapy	37
Atrx	31
1p/19q	25

Discussion

COVID-19 Pandemic and Funding Trends

The COVID-19 pandemic has had a significant impact on the field of oligodendroglioma research, as evident from our bibliometric analysis [13-15]. One notable effect was the decrease in funding during the pandemic. The year-on-year trends in funding showed a substantial peak in 2014, followed by a sharp decline in funding from 2020 to 2022. This decrease in financial support likely affected the overall productivity of research, as demonstrated by a decline in the total number of articles published during the same period. The reduction in funding and research output highlights the importance of robust funding mechanisms to sustain scientific progress, especially during challenging times such as a global pandemic.

Global Inequity

Our analysis revealed significant global inequities in the quantity of oligodendroglioma research publications. Researchers from HICs dominated the publication landscape. Researchers from LMICs also contributed to the literature. However, researchers from low-income economies were notably underrepresented in the publications. This disparity in representation suggests that resources, infrastructure, and research opportunities in HICs have provided a conducive environment for oligodendroglioma research [16]. In contrast, researchers from LMICs may face greater challenges in conducting and disseminating their research, emphasizing the need for global collaborative efforts to bridge this gap and promote equitable contributions from researchers worldwide.

Collaboration Trends and Gender Inequity

We identified extensive collaboration among authors in the field of oligodendroglioma research. The network visualizations revealed 23 clusters with a substantial number of collaborative links, indicating a highly interconnected research community. It is known that collaborations between researchers can facilitate knowledge exchange, foster innovative ideas, and enhance the quality and impact of research [17-19]. However, we also noted gender inequity within this collaborative network. Out of the 731 researchers whose gender could be estimated, a significant majority (78.7%) were male, while only 21.3% were female researchers. This gender disparity may reflect broader issues within the scientific community and merits attention to create a more inclusive and diverse research environment [20].

Top Keywords

The analysis of the most frequently used keywords in oligodendroglioma research provides valuable insights into the current focus and trends within the field. Prognosis emerges as the most prominent keyword, indicating a significant interest in understanding the factors that influence patient outcomes. Identifying prognostic markers is crucial for tailoring treatment strategies and improving overall survival rates [21]. Further research in this area can lead to the development of risk stratification models, enabling early interventions and personalized therapies [22]. Notably, the keyword "IDH" features prominently, reflecting a growing focus on investigating the role of isocitrate dehydrogenase mutations in oligodendroglioma pathogenesis. These mutations are considered defining molecular markers in these tumors and hold potential implications for targeted therapies [23,24]. Understanding the molecular mechanisms driven by IDH mutations could open new avenues for precision medicine interventions, offering more effective and personalized treatment options [25].

The emphasis on therapy-related keywords, such as chemotherapy and temozolomide, underscores the ongoing efforts to explore treatment options for oligodendroglioma patients [26,27]. The inclusion of radiotherapy as a prominent keyword highlights the significance of multimodal treatment approaches in managing these tumors effectively [28]. As therapeutic advancements continue, integrating targeted therapies and immunotherapies may further enhance treatment responses and patient outcomes [25]. Furthermore, investigations into combination regimens and their impact on the tumor microenvironment are essential for overcoming therapeutic resistance [29]. Addressing challenges related to resistance mechanisms will be crucial in developing durable and personalized treatments for patients with oligodendroglioma [30-32].

Molecular characteristics and genetics are central themes in oligodendroglioma research, reflecting their critical role in shaping current investigations. Immunohistochemistry emerges as a significant keyword, indicating a focus on investigating the molecular characteristics of oligodendroglioma through immunological markers [24]. Understanding these markers can aid in accurate diagnosis and facilitate potential targeted therapies. Additionally, specific genes and chromosomal alterations, such as "Atrx" and "1p/19q," are of paramount importance in oligodendroglioma research [33,34]. Exploring these genetic aspects is crucial for comprehending tumor development and progression, presenting essential targets for further investigation. By directing research efforts toward these key themes, researchers can advance our understanding of oligodendroglioma, develop innovative treatment strategies, and ultimately improve patient outcomes.

## Conclusions

This comprehensive bibliometric analysis of oligodendroglioma research has revealed several significant themes and implications for the scientific community. The analysis highlighted the impact of the COVID-19 pandemic on research funding and publication trends, underscoring the need for resilient funding mechanisms to sustain scientific progress during challenging times. Global and gender inequities in oligodendroglioma research were evident, emphasizing the importance of fostering collaboration and inclusivity among researchers worldwide. The highly interconnected research community identified through collaboration analysis suggests the potential for productive knowledge exchange and innovation in the field. Furthermore, the emphasis on specific keyword trends, such as genetic aberrations, and molecular characteristics highlights promising areas for further investigation, with the potential to advance our understanding of oligodendrogliomas and improve patient outcomes. By addressing these, the scientific community can collectively work toward better management and treatment strategies ultimately benefitting patients and advancing neuro-oncological research as a whole.
